# Early visual processing relevant to the reduction of adaptation-induced perceptual bias

**DOI:** 10.1038/s41598-021-94091-x

**Published:** 2021-07-29

**Authors:** Tomokazu Urakawa, Motoyoshi Tanaka, Yuta Suzuki, Osamu Araki

**Affiliations:** Department of Applied Physics, Faculty of Science, Tokyo University of Science, 6-3-1 Niijyuku, Katsushika-ku, Tokyo, 125-8585 Japan

**Keywords:** Cognitive neuroscience, Perception

## Abstract

Visual perception is biased by the preceding visual environment. A well-known perceptual bias is the negative bias where a current percept is biased away from the preceding image (adaptor). The preceding adaptor induces augmentation of early visual evoked potential (the P1 enhancement) of the following test image; the adaptor may invoke certain visual processing for the subsequent test image. However, the visual mechanism underlying P1 enhancement remains unclear. The present study assessed what the P1 alteration reflects in relation to the occurrence of the negative bias. In terms of inter-individual differences, we report that the P1 enhancement of the Necker lattice significantly correlated with the reduction of the reverse-bias effect. Further analyses revealed that the P1 enhancement was insusceptible to neural adaptation to the adaptor at the level of perceptual configuration. The present study suggests that prolonged exposure to a visual image induces modulatory visual processing for the subsequent image (reflected in the P1 enhancement), which is relevant to counteraction of the negative bias.

## Introduction

Perception of a visual scene does not exclusively arise based on the scene itself and instead depends on the preceding visual environment. There is a negative bias, called the adaptation aftereffect^1–3^, in which prolonged exposure to a certain visual image (i.e., an adaptor lasting for several seconds or minutes) biases the perception of a following test image away from the former in a certain feature dimension ranging from rudimental features, such as an orientation of a bar (the tilt aftereffect)^4^, to more complex objects like facial categories^5^. The negative bias was posited to function in the uptake of conspicuous (or new) visual properties in relation to the preceding visual environment to which the visual system is exposed^3,6^.

Neural activities relevant to the negative biases have been reported. Previous studies using functional magnetic resonance imaging (fMRI) showed that when the adaptor induced aftereffects for a subsequent test image, neural adaptation (reduction of neural activity) for the test image occurred at visual areas responsive to features/categories of the adaptor^7–9^. Electroencephalographic (EEG) studies also reported that the category-specific adaptation aftereffect was reflected in neural adaptation in N170 (visual evoked potential, VEP, at a latency of around 170 ms) to the test image, which was consistent with the preceding adaptor in image categories^10^; this suggests that the visual process related to the negative bias starts at an early stage of visual processing. Such neural adaptation to the adaptor has been argued to underlie the emergence of the negative bias.

In previous EEG studies of the negative bias regarding images of the human face^10–12^, presentation of an adaptor induced P1 enhancement at a latency of around 100–150 ms (a VEP component preceding N170) for a subsequent test image. Similarly, another previous EEG study revealed that prolonged exposure to visual motion increased the P1 amplitude of the following motion test stimulus, although P1 was not focused on^13^. Although neural adaptation to a test stimulus under emergence of the negative bias was reported to occur when a feature/category of the adaptor was the same as that of the test stimulus, as described above, the P1 enhancement for the test stimulus appeared to occur regardless of such consistency between the adaptor and the test stimulus^10,13^. As stated previously^12^, it is thus possible that prolonged exposure to the preceding visual stimulus invokes or promotes different visual processing for the following visual stimulus regardless of the category or content of the stimulus. Such visual processing is likely based on a mechanism not directly related to the generation of the negative bias and may be reflected in the P1 enhancement. To our best knowledge, the P1 alternation for a visual image, induced by a preceding adaptor, has not been a focus of interest, and little is known about what the P1 enhancement induced by the adaptor reflects and its relationship with the negative bias for the visual image.

Another perceptual bias (other than the negative bias) is the positive bias, which has been investigated recently in terms of serial dependence^14^. In the positive bias, the current percept of an image is attracted toward a prior image presented for a short period of time, typically up to around 500 ms in numerous feature dimensions from grating orientation^14,15^ to higher properties such as face identity^16^ or numerosity^17–19^. A suggested goal of the positive bias was to stabilize what we see over time in the face of an ever-fluctuating sensory environment^14,20^. For the positive bias, a widespread cortical network consisting of visual and fronto-parietal areas, including areas related to working memory, was reported to be involved^9^, and neural representation relevant to the positive bias was observed in the primary visual cortex^21^. In a recent EEG study using a neural decoding analysis^19^, it was argued that the neural signature relevant to the positive bias started to appear at as early as around 60 ms following the onset of an image for which the positive bias was expected. The perceived visual world is inevitably shaped through the negative bias and positive bias, and the visual system delicately balances between the opposite biases to achieve their respective goals. These opposing biases were reported to be simultaneously present^14^ and each perceptual report is permeated by these biases even in an identical method of visual stimulation^22^. However, as the previous neuroimaging studies dealing with the negative or the positive bias evaluated neural activities related to the opposing biases perpendicularly, it remains unclear how the brain balances between neural processes in a direction of the positive bias and those in a direction of the negative bias through hierarchical visual processing until a visual percept is reached.

In the present study, we hypothesized that visual processing underlying the P1 enhancement reflects modulatory early neural processing relevant to such a balancing act under an experimental condition where the negative bias occurs. In particular, we assessed whether the P1 enhancement is related to reduction of the negative bias. A bistable image, such as a Necker cube^23^, is an ambiguous stimulus in the sense that the image on the observer’s retina is equally compatible with at least two possible perceptual interpretations. During prolonged observation of the bistable stimulus, the observers’ perception is only transiently stable and eventually reverses repeatedly between the two most probable interpretations (e.g., Refs.^2,24^). By utilizing this stimulus property of bistability, previous behavioral studies evaluated the effects of preceding stimulus history on a current percept of a bistable stimulus^2,25–29^. We employed a bistable image, termed the Necker lattice^30^, to induce a negative bias for bistable perception, the reverse-bias effect (e.g., Refs.^2,28^); the reverse-bias effect has been argued to originate from neural adaptation to an unambiguous version of the bistable stimulus. First, we investigated whether the reverse-bias effect and P1 enhancement for the test image (Necker lattice) occur by prolonged exposure to the preceding adaptor (an unambiguous version of the Necker lattice). The strength of the reverse-bias effect and the magnitude of the P1 enhancement were then respectively quantified for each participant. Evaluation of the relationship between behavioral and neural data in terms of inter-individual differences is a powerful analytical approach to deduce the neural mechanisms underlying behavioral data^31^, which was employed in our previous VEP studies of bistable perception^32,33^. In terms of inter-individual differences, we performed correlation analyses between the behavioral and VEP data to clarify whether P1 enhancement is related to reduction of the reverse-bias effect (a negative bias).

## Methods

### Participants

Nineteen healthy volunteers (19 males, age 20–24 years, mean ± SD, 22.2 ± 1.17 years), all of whom were right-handed and had normal visual acuity, participated in this study. Informed consent was received from all participants. Data obtained for three participants were excluded from the analysis due to not performing the behavioral task based on the instruction or to not reporting any perceptual alternation of the bistable image (Necker lattice) before the experiment. This study was approved by the ethics committee of Tokyo University of Sciences. All methods used in the present study were in accordance with the relevant guidelines and regulations.

### Stimulus and tasks

**Figure 1:**
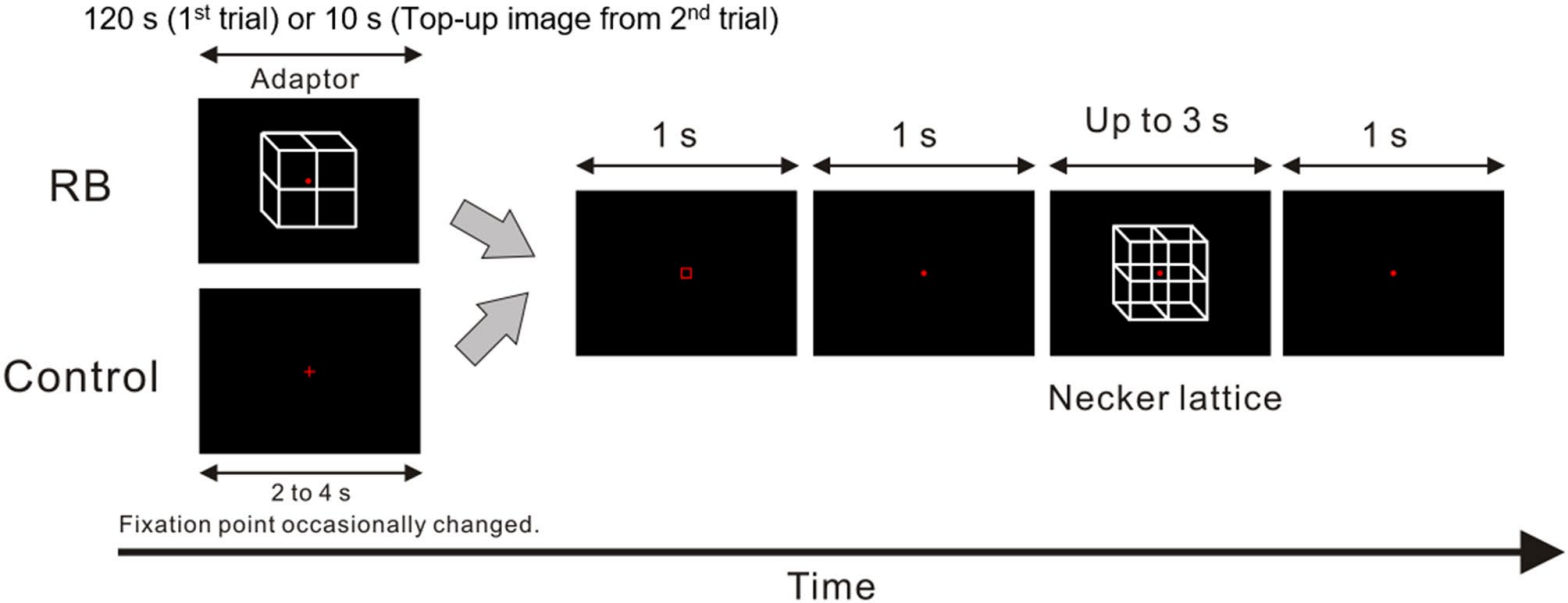
Time course of stimulus presentation in one trial. The stimulation scheme for one trial for both the reverse-bias (RB) condition and the Control condition is shown in figure. In the first trial of each session for the RB condition, an unambiguous adaptor (a lower-right-facing lattice) was initially presented for 120 s to induce the reverse-bias effect for a subsequently presented Necker lattice. Participants were asked to respond to a change in the fixation point during the presentation of the adaptor to maintain fixation on the adaptor. They were then required to report an initial perceived facing-orientation of the Necker lattice following its onset. From the second to the last trial in each session, the adaptor appeared for 10 s at the beginning of each trial to prevent reduction of the reverse-bias effect throughout the session (this shortly-presented adaptor was set as the top-up image). In the Control condition, the adaptor and the top-up image were not presented (see “Methods” for details).

Visual images were presented on a liquid crystal display (BENQ XL2540) at a refresh rate of 240 Hz. The presentations of the visual images were controlled by the MATLAB Psychophysics Toolbox^34,35^. The experimental procedure for one trial of each of the two experimental conditions is shown in Fig. 1. The reverse-bias effect was expected in the Reverse-bias condition (this condition is hereafter referred to as the RB condition) and the reverse-bias effect was not expected in the Control condition. All participants initially underwent the Control condition to prevent the reverse-bias effect induced in the RB condition from carrying over into the Control condition; the first trial of the RB condition started after finishing all trials of the Control condition.

In the RB condition, the lower-right facing lattice (the adaptor) with a red fixation point was initially presented at the center of the screen for 120 s for the first trial. The luminance at the background was 0.05 cd/m^2^. The size and mean luminance of the adaptor were 2.6° × 2.6° and 2.08 cd/m^2^, respectively. An initial percept of the Necker lattice is likely to be a lower-right-facing lattice without the adaptor, which can be accounted for by “the view/light from above prior” in shaping a visual percept^36–38^. Due to this, the present study did not use the upper-left-facing lattice as the adaptor to preclude the ceiling effect of the reverse-bias effect. During the presentation period of the adaptor, the fixation point occasionally changed to the red cross for 250 ms. The mean number of fixation changes during presentation of an adaptor was 28.46 ± 0.02 (SD) across participants. The time interval between two consecutive fixation changes was set at more than 1 s. Participants were asked to look at the fixation point, and to respond to the change as quickly and accurately as possible by pressing the response button (an up arrow key) in front of them. This behavioral task was included to prevent participants from paying attention to a certain feature of the adaptor to intentionally shape a percept of the following Necker lattice; this may affect emergence of the reverse-bias effect. Following the adaptor, the blank image with the red square frame and that with the fixation point immediately and consecutively appeared in this order for 1 s each. The presentation of the square frame was set to indicate the end of the detection task of the fixation change. Presentation of the square frame was also expected to equally control for participants to anticipate the timing of the forthcoming Necker lattice under both the RB and the Control conditions; the time interval between the presentation of the rectangle and the Necker lattice was identical across conditions. The Necker lattice (a bistable image that can induce either a lower-right-facing or upper-left-facing lattice) was then presented. The mean luminance of the Necker lattice was 3.24 cd/m^2^. To effectively induce the reverse-bias effect, the size of the Necker lattice was the same as that of the preceding adaptor, and the spatial position at which the Necker lattice was presented was identical to that at which the adaptor was presented. When the Necker lattice was presented, participants were asked to report a current percept of the Necker lattice by pressing the response button in front of them; pressing the left arrow key for a percept of the upper-left-facing lattice and the right arrow key for a percept of the lower-right-facing lattice. The Necker lattice continuously appeared up to 3 s unless the participants reported its initial percept. When the perceptual report was obtained, the presentation of the Necker lattice automatically ceased. A blank image with the fixation point lasted for 1 s between trials. Based on a previous behavioral study^39^, a top-up image was employed to avoid reduction of the reverse-bias effect throughout the trials. At the beginning of each trial from the second to the last, the top-up image identical to the adaptor used in the first trial was instead presented for 10 s. The spatial position at which the top-up image was presented was the same as that at which the adaptor was presented. The fixation point of the top-up image occasionally changed to the red cross, as in the adaptor of the first trial, and the participants were asked to respond to the change as in the task during the presentation period of the adaptor. The mean number of fixation changes during presentation of a top-up image was 1.41 ± 0.11 (SD) across participants. The scheme of image presentation was identical to that of the first trial after presentation of the top-up image.

In the Control condition, the adaptor and the top-up stimulus did not appear, but a blank image lasting for 2–4 s (randomly selected) was instead presented prior to the presentation of the blank image with the red square frame. The fixation point of the blank image occasionally changed, similar to the adaptor/top-up image in the RB condition. The mean number of fixation changes during presentation of a blank image was 0.37 ± 0.02 (SD) across participants. The scheme of image presentation in the Control condition was identical to that in the RB condition except for the images presented prior to the presentation of the red square frame (see Fig. 1). The behavioral task in the Control condition was the same as that in the RB condition. Each condition consisted of 120 trials divided into 6 sessions. In the RB condition, the adaptor lasting for 120 s was presented for the first trial of each session. Participants were allowed to rest between sessions if needed.

### Analysis of behavioral data

In each condition, we first excluded the trials in which participants did not report the perceived orientation of the Necker lattice. For the remaining trials, the trials in which the response time to reporting the perceived orientation of the Necker lattice exceeded 200 ms were further selected. After this selection of trials, the mean number of remaining trials was 119.50 ± 0.97 (SD) (117 at minimum, 120 at maximum) for the RB condition and 118.94 ± 2.21 (SD) (113 at minimum, 120 at maximum) for the Control condition. We then calculated the proportion of the selected trials in which participants reported a left-facing percept toward the Necker lattice. Separately, the trials were then selected on the basis that participants responded to all of the fixation change(s) within 1 s following each of the changes. The ratio of these trials to all trials with fixation changes was then calculated in each condition.

### EEG recording

Neural activity in the RB and Control conditions was recorded by an electroencephalography (EEG) processor with 57 electrodes on the scalp (EEG-1200, Nihon Kohden, Tokyo, Japan; EasyCap GmbH, Herrsching, Germany). Impedance at each electrode was kept lower than 10 kΩ. EEG signals were digitized at 1 kHz and recorded with a 0.5–300-Hz band-pass filter. For data acquisition, EEG signals were referenced to the right earlobe.

### Analysis of EEG data

EEG signals were band-pass filtered offline at 0.5–30 Hz, and EEG epochs from 200 ms before to 500 ms after the onset of the Necker lattice were collected. We calculated the mean of the EEG epochs across trials to obtain VEPs time-locked to the Necker lattice. In this calculation, EEG epochs in the trials that were not defined as valid (see the section of analysis of behavioral data) were omitted. Then, the remaining EEG epochs containing a deflection of greater than ± 100 μV in at least one electrode were then further excluded from averaging across trials in order to remove EEG signals containing artifacts. By this procedure, at least 112 artifact-free EEG signals were averaged in each condition for each participant. The mean amplitude for a period of − 200 to 0 ms relative to the stimulus onset was used as the baseline, and the VEP obtained was re-referenced to the nose tip. The present study defined the electrode regions of interest (ROI) for the left and right posterior. The electrodes used to determine the electrode ROI were O1, PO3, PO7, P3, P5, and P7 for the left posterior side, and O2, PO4, PO8, P4, P6, and P8 for the right posterior side. The areal mean of the VEPs was then calculated for each electrode ROI. The peak latency and peak amplitude for the areal mean were then assessed by repeated-measures two-way analysis of variance (ANOVA) with condition and electrode ROI factors. Based on previous studies^32,33,40^, the difference in amplitude between the RB and Control conditions was evaluated using a series of two-tailed *t*-tests through successive time points at the latency range of 90–400 ms. When the *t*-tests exceeded the 0.05 criterion for at least 20 subsequent time points, the amplitude difference between conditions was considered to be significant.

As in our previous studies^32,33^, the present study evaluated the relationship between behavioral and neural data in terms of inter-individual differences; this is a powerful analytical approach to deduce the neural mechanisms underlying behavioral data^31^. Specifically, to clarify whether P1 enhancement is related to reduction of the reverse-bias effect, we calculated Pearson’s correlation coefficients between the strength of the reverse-bias effect and the difference in peak latency/amplitude of VEPs (RB condition–Control condition) across participants; the strength of the reverse-bias effect was quantified by subtracting the proportion of the left-facing percept in the RB condition from that in the Control condition. P-values obtained in the correlation analyses were controlled by the false discovery rate (FDR: q = 0.05) based on a previous study^41^, and adjusted p-values are expressed as the *adj_p* hereafter.

In further EEG analysis, we sorted EEG epochs based on perception of the Necker lattice and then calculated VEP time-locked to the Necker lattice in each percept for each condition. This analysis was performed to evaluate whether P1/N1 reflects neural adaptation to the adaptor at the level of 3D lattice configuration and whether the current experiment can invoke the VEP component related to perceptual reversal, referred to as reversal negativity (e.g., Refs.^30,44^). Analytical procedures for calculating VEPs in this analysis were the same as those in the former VEP analysis except for the sorting of EEG epochs based on perceptual reports of the Necker lattice. For each VEP, at least 32 artifact-free EEG signals were averaged across participants. The peak latency and peak amplitude at each electrode ROI were submitted to repeated-measures three-way analysis of variance (ANOVA) with factors of perception for the Necker lattice, condition, and electrode ROI. The peak latency/amplitude, which were not unequivocally determined by visual inspection, was treated as missing data throughout all statistical analyses performed in this study. Data are shown as the mean ± SE except when noted otherwise.

## Results

### Behavioral data

**Figure 2:**
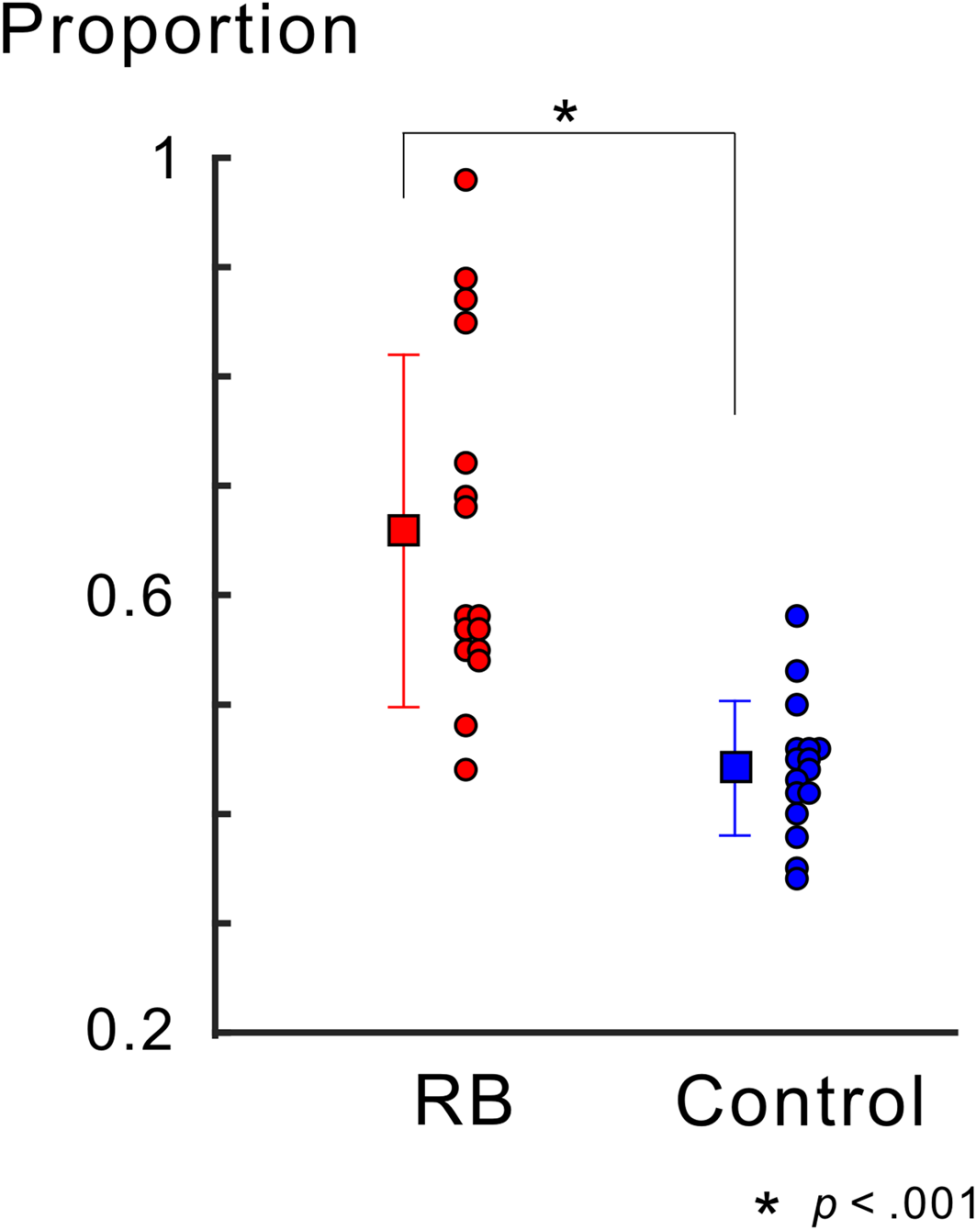
Proportion of trials in which a perceived facing-orientation of the Necker lattice was opposite to that of the adaptor. The proportions of trials in which the Necker lattice was perceived as an upper-left-facing lattice (opposite orientation to that of the adaptor) are shown for all participants. The mean proportion is indicated by a black square with ± SD. The proportion in the RB condition was significantly higher than that in the Control condition. Some individual data points are horizontally shifted for display purposes. As in the other figures, data for the RB condition and that for the Control condition were depicted in red and in blue, respectively.

Participants were instructed to respond to any fixation change preceding the image of the red square frame in both the RB and Control conditions. The response rate to all fixation changes was nearly or equal to 1 for all participants in both the RB and Control conditions, suggesting that the participants successfully performed the task to detect the fixation change. Regarding the behavioral task for the Necker lattice, the mean rate of reporting a perceived orientation of the Necker lattice was also nearly or equal to 1 across participants in both conditions. The proportion of trials in which participants reported that the Necker lattice was perceived as an upper-left-facing lattice is shown in Fig. 2. The mean proportion of the left-facing percept was significantly higher in the RB condition than in the Control condition (paired *t*-test, *t* (15) = 5.689, *p* < 0.001), suggesting that the reverse-bias effect occurred in the RB condition. The mean response time (RT) to the Necker lattice in the RB condition did not significantly differ from that in the Control condition (for the RB condition, 1119.46 ± 237.95 (SD) ms; for the Control condition, 1183.99 ± 80.21 (SD) ms; paired *t*-test, *t* (15) = 1.093, *p* = 0.292).

### EEG data

**Figure 3:**
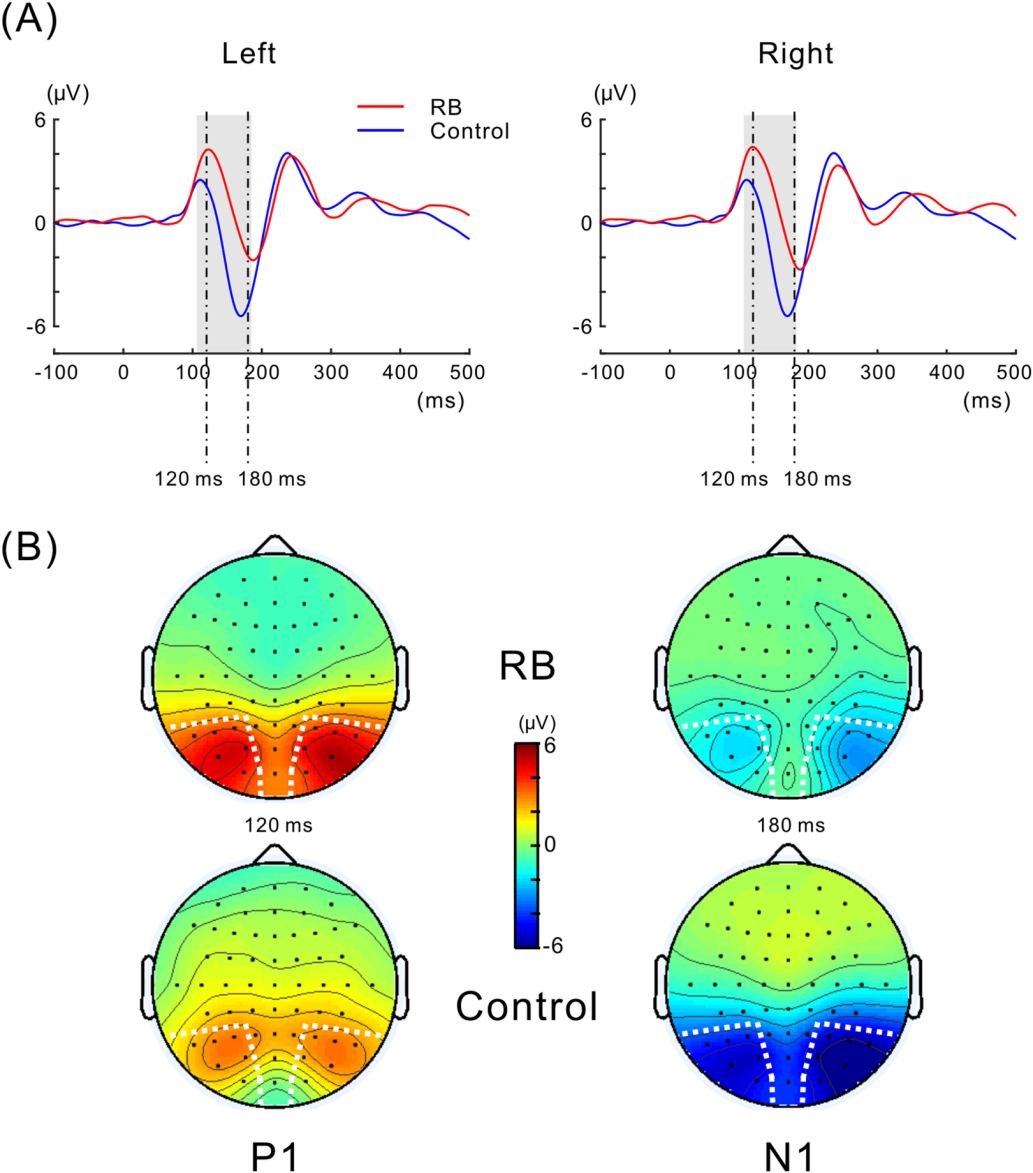
VEPs to the Necker lattice in the RB and Control conditions. (**A**) Grand-averaged VEPs at the left and at the right electrode ROIs. VEP waveforms recorded in the RB condition are depicted as a continuous line and those recorded in the Control condition are depicted as a dotted line. P1 amplitude at a latency of around 120 ms increased in the RB condition compared with that in the Control condition, whereas the N1 amplitude at the latency of around 180 ms decreased in the RB condition. The time interval in which there was a significant difference in VEP amplitude between the conditions is shaded in gray (for the procedures of the statistical analyses, see “Methods”). (**B**) Isocontour maps at latencies of 120 ms and 180 ms for the RB condition and Control condition. Both P1 (left panel) and N1 (right panel) were prominent at the posterior electrode sites in both conditions. Electrodes selected to define the electrode ROI were enclosed with a white dotted line for the left and the right side.

Grand-averaged VEPs time-locked to the onset of the Necker lattice are presented in Fig. 3A. In both the left and right electrode ROIs, P1 and N1 components clearly emerged across the RB and Control conditions. Isocontour maps for each condition at a representative latency of P1 (120 ms) and N1 (180 ms) are shown in Fig. 3B. In both the RB and Control conditions, the most prominent P1 and N1 components were all spatially covered by the electrode ROIs (see “Methods” section for the electrode ROIs).

**Figure 4:**
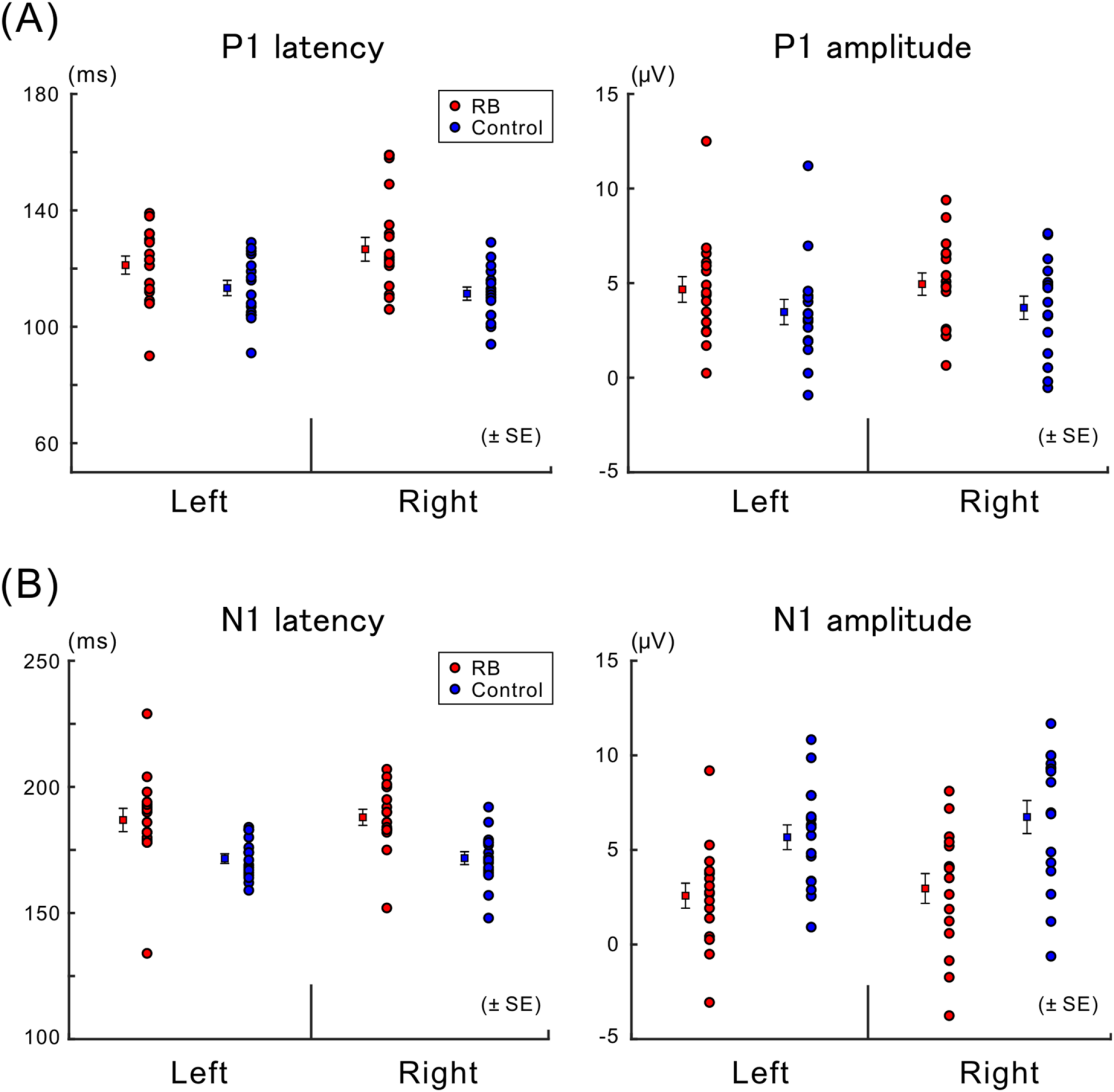
Latencies and amplitudes of VEPs. (**A**) Mean of peak latencies (left) and mean of peak amplitudes (absolute values) (right) for P1 with their respective means are illustrated across participants. The P1 latency in the RB condition was significantly longer than that in the Control condition (*p* < 0.001), and the P1 amplitude in the RB condition was significantly larger than that in the Control condition (*p* < 0.006). (**B**) Mean of peak latencies (left) and mean of peak amplitudes (absolute values) (right) for N1 with their respective means are illustrated across participants. The N1 latency in the RB condition was significantly shorter than that in the Control condition (*p* < 0.001), whereas the N1 amplitude in the RB condition was significantly smaller than that in the Control condition (*p* < 0.001).

The peak P1 latencies/amplitudes (absolute values) across participants with their respective means are shown in Fig. 4A. The peak latency of P1 significantly increased in the RB condition compared with the Control condition (main effect of condition: *F* (1, 15) = 41.139, *p* < 0.001). There was no significant difference in the peak latency between the left and right electrode ROIs (main effect of electrode ROI: *F* (1, 15) = 0.443, *p* = 0.516), and the interaction between these factors for the latency was also not significant (*F* (1, 15) = 2.422, *p* = 0.140). As for the peak amplitude of P1, we noted a significant increase in the RB condition compared with the Control condition (main effect of condition: *F* (1, 15) = 10.083, *p* = 0.006), but there was no significant difference between the left and right electrode ROIs (main effect of electrode ROI: *F* (1, 15) = 0.447, *p* = 0.514). Enhancement of the first positive VEP component, which was induced by the adaptor, was consistent with previous VEP studies focusing on the face-related aftereffects (e.g., Refs.^10,11^). The relative increase in the P1 amplitude in the RB condition compared with the Control condition was comparable between the left and right electrode ROIs (the interaction between the two factors for P1 amplitude was also not significant; *F* (1, 15) = 0.044, *p* = 0.837).

As illustrated in Fig. 3A, positive enhancement for the RB condition over the Control condition was continuously observed from the latency range of P1 to that of N1. Two-tailed *t*-tests, successively performed for each consecutive time point (see “Analysis of EEG data” in the “Methods” section), revealed that the positive shift of VEP was significant for the left and right electrode ROIs (for the left electrode ROI, *ps* < 0.05 at latencies of 106–185 ms; for the right electrode ROI, *ps* < 0.05 at latencies of 107–183 ms). At later latencies up to 500 ms, there was no significant VEP shift between conditions for both left and right electrode ROIs.

The peak N1 latencies/amplitudes across participants with their respective means are shown in Fig. 4B. The peak latency of N1 significantly increased in the RB condition compared with the Control condition (main effect of condition: *F* (1, 15) = 49.223, *p* < 0.001). There was no significant difference in the latency between the left and right electrode ROIs (main effect of electrode ROI: *F* (1, 15) = 0.034, *p* = 0.857), and the interaction between these factors in the latency was also not significant (*F* (1, 15) = 0.037, *p* = 0.850). The amplitude of N1 significantly decreased in the RB condition compared with the Control condition (main effect of condition: *F* (1, 15) = 103.116, *p* < 0.001), but there was no significant difference in the amplitude between the left and right electrode ROIs (main effect of electrode ROI: *F* (1, 15) = 3.268, *p* = 0.091). The diminished and delayed N1 toward the Necker lattice is consistent with a previous study on the adaptation aftereffect for face/hand images^10^. In our experiment, local and overall configurations of the adaptor and Necker lattice were physically similar. It is therefore possible that neural adaptation for the Necker lattice occurred for N1 to some extent. Further statistical analysis demonstrated a significant interaction between condition and electrode ROI: *F* (1, 15) = 9.691, *p* = 0.007); as shown in Fig. 4B, the relative decrease in N1 in the RB condition compared with the Control condition for the right electrode ROI was slightly larger in magnitude than that for the left electrode ROI. Although the N1 amplitude in the RB condition was significantly smaller than that in the Control condition for each electrode ROI, the N1 amplitude in the left electrode ROI did not significantly differ from that for the right electrode ROI in each condition (post-hoc tests with a Bonferroni correction, overall α = 0.05).

### Correlation between behavioral data and VEPs

**Figure 5:**
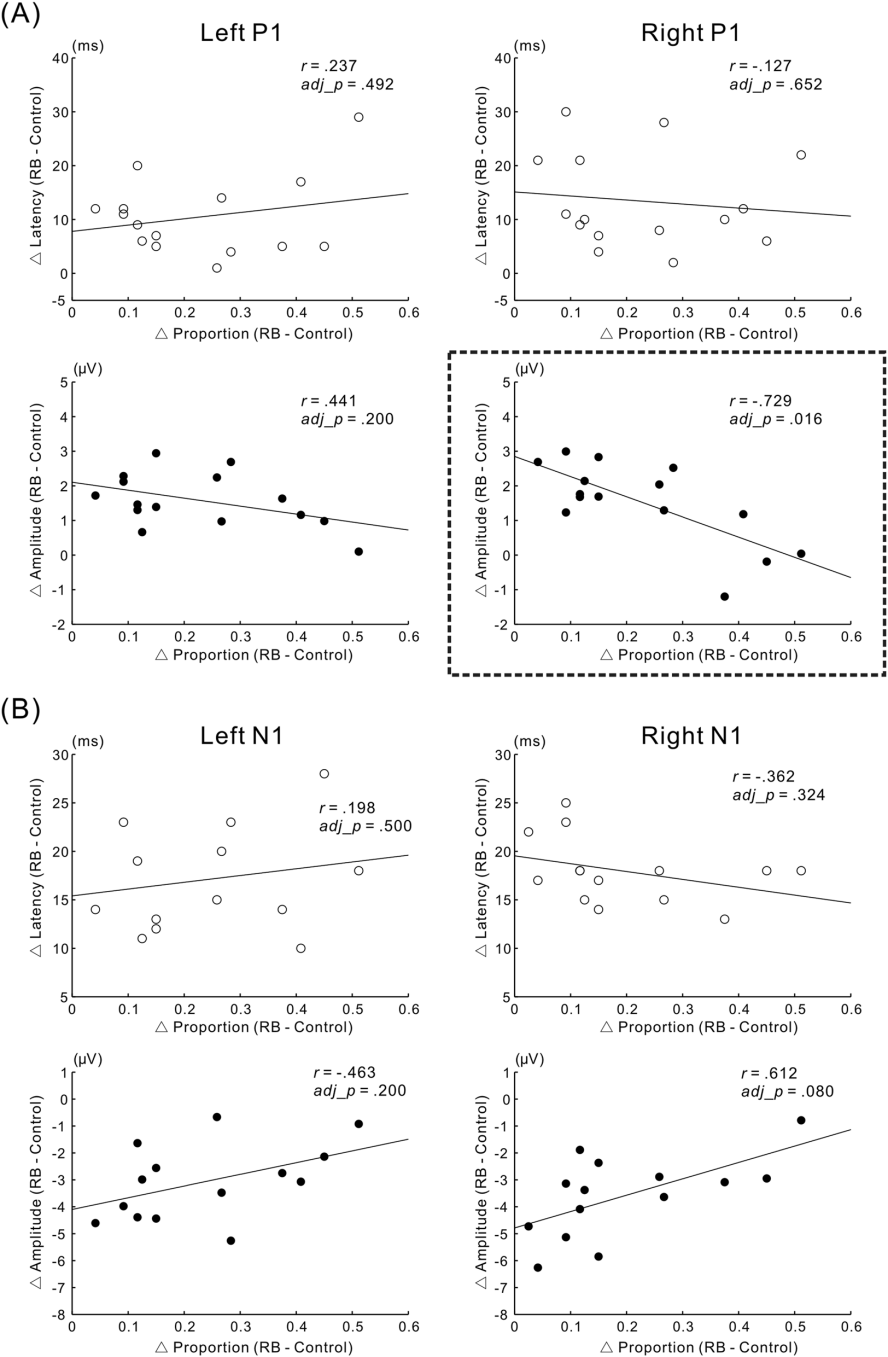
Relationship between VEPs and the strength of the reverse-bias effect across participants. The ordinate indicates the difference in latency or amplitude (RB–Control). The abscissa indicates the strength of the reverse-bias effect, which was quantified by subtracting the proportion of trials with the Necker lattice being perceived as an upper-left-facing lattice (opposite to the facing-orientation of the adaptor) in the Control condition from that in the RB condition. Significance was controlled by the false discovery rate (FDR: q = 0.05) throughout all correlation analyses. (**A**) The correlation between P1 and the strength of the reverse-bias effect for the left electrode ROI (left panel) and the right electrode ROI (right panel). For the right electrode ROI, there was a significant correlation between the relative enhancement of P1 in the RB condition compared with the Control condition and a decrease in the strength of the reverse-bias effect. The scatter diagram with a significant correlation is enclosed by a squared dotted line. (**B**) As for N1, there was no significant correlation between latency or amplitude and the strength of the reverse-bias effect for each electrode ROI.

The present study evaluated whether the latency/amplitude of P1 and N1 is correlated with the strength of the reverse-bias effect across participants. Behavioral and VEP data with outliers were excluded before performing the correlation analyses; outliers were defined as values exceeding the mean ± 2 SD. Correlation analysis for P1 at the left electrode ROI revealed no significant relationship between latency/amplitude and the strength of the reverse-bias effect (*r* = 0.237, *adj_p* = 0.492 for latency; *r* = − 0.441, *adj_p* = 0.200 for amplitude) (left panel of Fig. 5A). Regarding P1 at the right electrode ROI, we found a significant relationship between increased amplitude and reduction of the perceptual bias (*r* = − 0.729, *adj_p* = 0.016), whereas latency did not correlate with the strength of the bias (*r* = − 0.127, *adj_p* = 0.652) (the right panel of Fig. 5A). This significant relationship between alteration of P1 and the decrease in the strength of the reverse-bias effect is consistent with our current hypothesis. As for N1, there was no significant correlation between the latency/amplitude and the strength of the reverse-bias effect at the left electrode ROI (*r* = 0.198, *adj*_*p* = 0.569 for latency; *r* = 0.463, *adj_p* = 0.200 for amplitude) (left panel of Fig. 5B) or the right electrode ROI (*r* = − 0.362, *adj_p* = 0.324 for latency; *r* = 0.612, *adj_p* = 0.080 for amplitude) (right panel of Fig. 5B).

### Evaluating neural adaptation and the emergence of reversal negativity

As described above, behavioral data demonstrated that the reverse-bias effect significantly occurred in the RB condition. Based on previous psychological studies^28,29,42^, the neural mechanism underlying the reverse-bias effect was posited to be neural adaptation to a precedingly presented unambiguous version of a bistable stimulus. Therefore, the present study further evaluated whether P1 and N1 time-locked to the onset of Necker lattice reflect neural adaptation to the adaptor at the level of 3D lattice configuration by sorting trials based on perceptual reports of a Necker lattice (percept of an upper-left- facing lattice or that of a lower-right-facing lattice; hereafter, we referred to the former as Percept-L and the latter as Percept-R). When neural adaptation occurred, it was predicted, in the RB condition, that the neural adaptation will induce VEP related to a configuration of the lower-right-facing lattice (VEP calculated with trials of Percept-R) in comparison with VEP related to a configuration of the upper-left-facing lattice (VEP calculated with trials of Percept-L); the adaptor was a lower-right-facing-lattice. On the other hand, such VEP diminishment due to neural adaptation was not expected in the Control condition; there was no adaptor in the Control condition.

**Figure 6:**
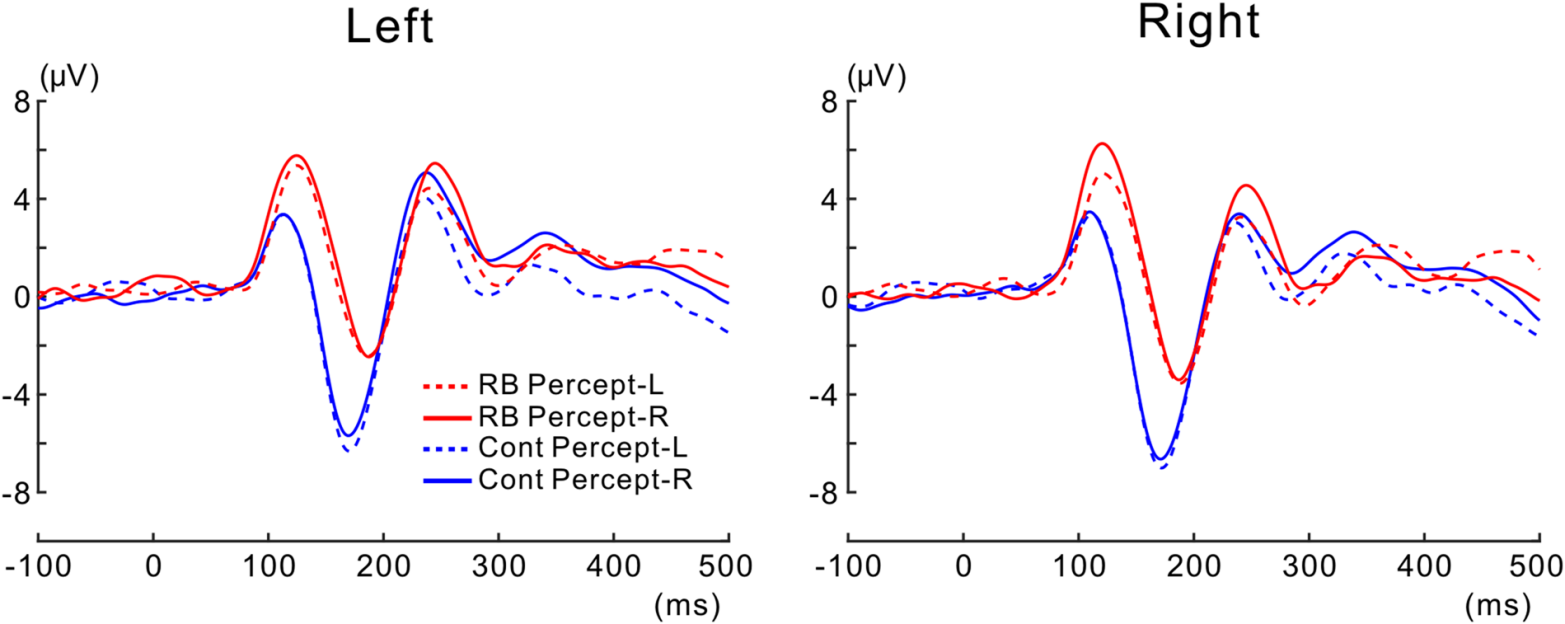
VEPs sorted with each percept for the Necker lattice. Grand-averaged VEPs calculated with trials where the Necker lattice was perceived as an upper-left-facing lattice (Percept-L) and those calculated with trials where the Necker lattice was perceived as a lower-right-facing lattice (Percept-R) are shown as a dotted line and continuous line, respectively. Grand-averaged VEP waveforms recorded in the RB condition are depicted in red and those recorded in the Control condition are depicted in blue. In each condition, the grand-averaged VEP for Percept-L was almost the same as that for Percept-R (see texts for details).

**Figure 7:**
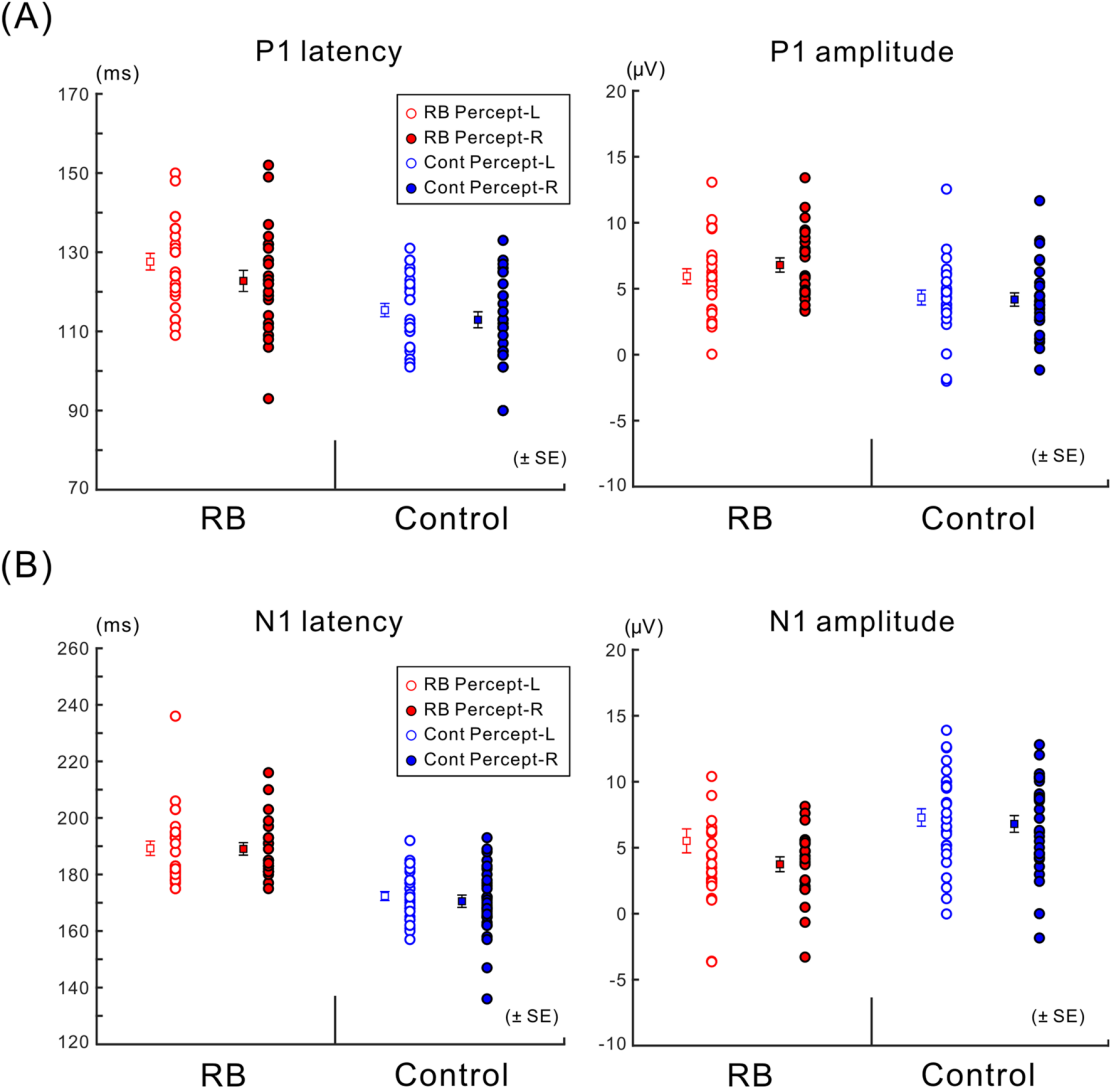
Latencies and amplitudes of VEPs for each percept of the Necker lattice. (**A**) Mean of peak latencies (left) and mean of peak amplitudes (absolute values) (right) for P1 with their respective means are illustrated across participants. The peak latency and peak amplitude of P1 were not significantly affected by any factor related to the experimental condition or perception toward the Necker lattice; these results thus supported that neural adaptation to the adaptor at the level of 3D lattice configuration did not affect P1 if present (see text for details). (**B**) Mean of peak latencies (left) and mean of peak amplitudes (absolute values) (right) for N1 with their respective means are illustrated across participants. The peak latency and peak amplitude of N1 were not significantly affected by any factor related to the experimental condition or perception toward the Necker lattice; these findings supported that N1 was not confounded by the neural adaptation at the level of 3D lattice representation or RN (reversal negativity, a negative-going VEP component related to the perceptual alternation of a bistable stimulus) if either or both were present (see text for details).

For each perceptual report (Percept-L or Percept-R) in each condition, grand-averaged VEPs and peak latencies/amplitudes (absolute values) of P1/N1 across participants with their respective means are shown in Figs. 6 and 7, respectively. Opposite to the prediction based on neural adaptation to the adaptor at the level of 3D lattice configuration, the peak amplitude of P1 for trials of Percept-R was slightly larger than that for trials of Percept-L in the RB condition, whereas the former was comparable with the latter in the Control condition. Statistical analyses revealed no significant effects for the peak amplitude of P1 (the interaction between percept and condition, *F* (1, 11) = 2.972, *p* = 0.113; the interaction among percept, condition and electrode ROI, *F* (1, 11) = 2.326, *p* = 0.155) and for the peak latency of P1 (the interaction between percept and condition, *F* (1, 11) = 1.224, *p* = 0.292; the interaction among percept, condition and electrode ROI, *F* (1, 11) = 0.065, *p* = 0.804). These findings did not support that P1 reflected neural adaptation to the adaptor at the level of 3D lattice configuration. As such, there were no other significant differences in relation to perceptual reports of the Necker lattice in the peak amplitude of P1 (main effect of the percept, *F* (1, 11) = 0.362, *p* = 0.559; the interaction between the percept and electrode ROI, *F* (1, 11) = 1.760, *p* = 0.211) or in the peak latency of P1 (main effect of the percept, *F* (1, 11) = 4.856, *p* = 0.050; the interaction between the percept and electrode ROI, *F* (1, 11) = 0.180, *p* = 0.680).

Furthermore, the peak amplitude of P1 was significantly larger for the RB condition than for the Control condition (*F* (1, 11) = 56.425, *p* < 0.001) and the peak latency of P1 was significantly longer for the RB condition than that for the Control condition (*F* (1, 11) = 32.313, *p* < 0.001). The P1 was not significantly affected by the electrode ROIs in peak amplitude (main effect of electrode ROI: *F* (1, 11) = 0.065, *p* = 0.804) or peak latency (main effect of electrode ROI: *F* (1, 11) = 0.181, *p* = 0.679), and there were no other significant interactions in relation to the electrode ROIs for the peak amplitude of P1 (the interaction between electrode ROI and condition, *F* (1, 11) = 0.462, *p* = 0.510) or the peak latency of P1 (the interaction between electrode ROI and condition, *F* (1, 11) = 1.879, *p* = 0.198). These results were consistent with the P1 results reported above.

Regarding N1, in the RB condition, there was another factor that may have affected N1 in addition to neural adaptation to the adaptor. As the adaptor was a lower-right-facing lattice, perceptual alternation may have occurred when the following Necker lattice was perceived as an upper-left-facing lattice. In this case, VEP time-locked to the onset of the Necker lattice may have been confounded to some extent with another negatively-going VEP component related to perceptual alternation, reversal negativity (RN) (e.g., Refs.^30,43^). These two factors (i.e., the neural adaptation to the adaptor at the level of 3D lattice configuration and the perceptual alternation) will respectively give rise to a relative increase in N1 obtained with the trials of Percept-L in comparison with N1 obtained with the trials of Percept-R for the RB condition; these two factors were not expected to affect N1 in the Control condition. Statistical analysis revealed no significant interaction between the perceptual report for the Necker lattice and condition for the peak amplitude of N1 (*F* (1, 10) = 2.059, *p* = 0.484) or the peak latency of N1 (*F* (1, 10) = 0.383, *p* = 0.550). In addition, there was no significant interaction among the percept, condition, and electrode ROI in the peak amplitude of N1 (*F* (1, 10) = 0.016, *p* = 0.902) or peak latency of N1 (*F* (1, 10) = 0.796, *p* = 0.393). Thus, this study did not support that N1 was confounded by the neural adaptation at the level of 3D lattice configuration and/or with the RN.

Further statistical analyses revealed no other significant differences in relation to perceptual reports of Necker lattice for the peak amplitude of N1 (main effect of the percept, *F* (1, 10) = 0.772, *p* = 0.400; the interaction between the percept and electrode ROI, *F* (1, 10) = 0.723, *p* = 0.415) or for the peak latency of N1 (main effect of the percept, *F* (1, 10) = 0.066, *p* = 0.803; the interaction between the percept and electrode ROI, *F* (1, 10) = 1.326, *p* = 0.276). Similar to the N1 results reported above, the peak amplitude of N1 markedly decreased in the RB condition compared with the Control condition (*F* (1, 10) = 52.206, *p* < 0.001) and the peak latency of N1 was longer for the RB condition than for the Control condition (*F* (1, 10) = 123.531, *p* < 0.001) (Figs. 6 and 7B). The peak amplitude of N1 for the right electrode ROI was significantly larger than that for the left electrode ROI (the right electrode ROI, 6.069 ± 0.541 µV; the left electrode ROI, 5.064 ± 0.446 µV; main effect of electrode ROI, *F* (1, 10) = 5.146, *p* = 0.047), whereas the peak latency of N1 for the right electrode ROI did not significantly differ from that for the left electrode ROI (the right electrode ROI, 176.46 ± 3.76 ms; the left electrode ROI, 175.79 ± 3.92 ms; main effect of electrode ROI, *F* (1, 10) = 0.365, *p* = 0.559). Consistent with the preceding N1 results, there was a significant interaction between the condition and electrode ROI for the peak amplitude of N1 (for the left electrode ROI, 3.327 ± 0.575 µV in the RB condition and 6.553 ± 0.530 µV in the Control condition; for the right electrode ROI, 4.042 ± 0.611 µV in the RB condition and 7.747 ± 0.722 µV in the Control condition; *F* (1, 10) = 5.635, *p* = 0.039). As for the peak latency of N1, there was no significant interaction between the condition and the electrode ROI (for the left electrode ROI, 189.70 ± 2.81 ms in the RB condition and 170.34 ± 1.99 ms in the Control condition; for the right electrode ROI, 188.62 ± 2.06 ms in the RB condition and 172.48 ± 1.94 ms in the Control condition; *F* (1, 10) = 0.085, *p* = 0.776). In addition, although the N1 amplitude in the RB condition was significantly smaller than that in the Control condition for each electrode ROI, the N1 amplitude in the left electrode ROI did not significantly differ from that for the right electrode ROI in each condition (post-hoc tests with a Bonferroni correction, overall α = 0.05), which were again all consistent with the preceding N1 results.

## Discussion

The present study tested a hypothesis that the P1 enhancement, invoked under emergence of the reverse-bias effect (a negative bias), reflects early visual processing relevant to reduction of the reverse-bias effect. In terms of interindividual differences in behavioral and neural data, our study revealed that the P1 enhancement was significantly correlated with the reduction of the reverse-bias effect. Further analyses demonstrated that the P1 enhancement did not reflect neural adaptation to the adaptor at the level of perceptual configuration. As for N1 (a VEP component following P1), its amplitude was not significantly correlated with the strength of the reverse-bias effect across participants. In addition, N1 significantly decreased following the adaptor, but this decrease did not reflect neural adaptation to the adaptor at the level of perceptual configuration. In support of our hypothesis, the present study suggested that the adaptor, inducing a negative bias, invokes specific visual processing for the subsequent test image as early as 120 ms (reflected in P1), which is related to counteracting the negative bias.

Neural adaptation to the adaptor, being relevant to induction of the reverse-bias effect, is expected to begin to occur at a certain stage of hierarchical visual processing. As described above, P1 and N1 were not susceptible to neural adaptation to the adaptor at the level of 3D lattice configuration. However, neural adaptation at a lower level of visual processing than the level of 3D lattice configuration was expected. Previous psychological studies supported this possibility by demonstrating that the reverse-bias effect becomes weaker or is almost abolished when the adaptor and following test stimulus differ in size or are presented at different retinal regions (e.g., Refs.^2,28,44^). Based on these previous findings, neural adaptation to the adaptor, relevant to induction of the reverse-bias effect, is likely to primarily occur at the early visual area, such as V1, where the retinotopy is clearly organized; such neural adaptation likely occurs at the level of localized portions of a configuration of visual object rather than at the level of 3D lattice configuration. Neural generators of P1 and N1 were reported to lie at the extrastriate cortex ^45^, and VEP preceding P1, which originates from V1^45–47^, was not clearly evoked under the current experiment (see Figs. 3 and 6). In addition, the current stimulation paradigm was not able to selectively induce neural adaptation at the level of localized portions of a visual object irrespective of its configuration. Considering these limitations, it remains unclear at which level of hierarchical visual processing neural adaptation relevant to induction of the reverse-bias effect occurred in the present study.

When a bistable image is intermittently presented, the VEP time-locked to the onset of the image was reported to be positively enhanced at a latency of around 130 ms when participants reported perceptual alteration of the image in relation to a percept for the preceding image^43,48–50^. This positive VEP enhancement related to perceptual alternation is referred to as the reversal positivity (RP). As perceptual alternation from before to after the onset of the Necker lattice must have occurred to some extent in the RB condition, the RP may have been at least partly invoked by the current paradigm of stimulation. However, we argue that P1 enhancement reported in the present study was not critically affected by the RP for the following reasons. First, the inter-stimulus interval (ISI) between the adaptor and Necker lattice for each trial in the RB condition (2 s) did not lie within the range of ISI (less than about 400 ms) at which the RP was evoked in most of previous studies^43,48,49^. Second, participants were asked to perform a behavioral task irrelevant to perceptual configuration of the adaptor in the current experiment, mitigating occurrences of perceptual alternation. Finally, as reported in the Results section, our current findings regarding P1 were not significantly affected by the Necker lattice percept in relation to the unambiguous configuration of the preceding adaptor. Taken together, it is unlikely that the P1 enhancement reported in the current study was confounded by the RP.

Allocation of spatial attention to a certain location within a bistable image was previously proposed to allow a preferentially processed feature at the attended location, thereby shaping interpretation of the feature as “nearest” or “in the foreground”^51,52^. When such attention to a part of the bistable image spatially shifts to another location on a bistable image, the attentional shift leads to a P1 increase, as suggested in previous VEP studies of spatial attention (e.g., Refs.^53,54^), and the shift of attention may yield perceptual alternation. In this perspective, we cannot exclude the possibility that the P1 enhancement for the Necker lattice in the RB condition compared with the Control condition was at least partly induced by a shift in spatial attention. In our current stimulation for the RB condition, the adaptor was a lower-right-facing lattice and the following Necker lattice was perceptually biased by the adaptor to be an upper-left-facing lattice, in which a shift in spatial attention from the lower-right foreground (the adaptor’s foreground) to the upper-left foreground (the perceptually-biased foreground of Necker lattice) was expected. In the Control condition, as the adaptor was not presented, there was no experimental setting to prompt a perceptual change in the foreground of the Necker lattice and the percept of the foreground of the Necker lattice was likely confined to a lower-right-facing lattice (see Fig. 2) due to “the view/light from above prior” (e.g., Refs.^36–38^). Considering these points, there may have been more frequent shifts in spatial attention on the Necker lattice in the RB condition than in the Control condition, which may have led to the greater P1 enhancement in the RB condition. However, such possible shifts in spatial attention did not significantly affect our results. If such shifts in spatial attention yielded the P1 enhancement, the P1 amplitude should have been higher in the RB condition than in the Control condition at the right electrode ROI (being contralateral to the biased perceptual foreground of the Necker lattice), and the P1 amplitude in the RB condition should have been lower than that in the Control condition at the left electrode ROI. However, P1 enhancement in the RB condition relative to the Control condition did not significantly differ between the left and the right electrode ROIs, demonstrating that the P1 enhancement observed in our current study cannot be simply accounted for by the shift of spatial attention.

When an unambiguous version of a bistable image is exposed only for several seconds or shorter, a subsequent percept of the bistable image is more likely to be the same as that of the preceding unambiguous image (emergence of the positive bias for the bistable visual stimulus) (e.g., Refs.^28,29^). The present study did not have an experimental condition to invoke the positive bias; in our experiment, an unambiguous version of the Necker lattice, preceding the Necker lattice, appeared for a much longer period than that used in the previous studies of the positive bias. As such, we cannot exclusively attribute visual processing underlying the P1 enhancement to the positive bias for the bistable stimulus. Indeed, the strength of the reverse-bias effect was positive for most participants or around zero (see data in Fig. 5a,b with respect to the abscissa), although P1 enhancement was relevant to reduction of the reverse-bias effect across participants. This point aside, our current method of stimulation may have tapped neural processing related to the serial dependence (a positive bias) to some extent. The serial dependence was reported to operate over successive spatial locations, which were attended by participants^14^, and even an unattended visual feature within an attended location is expected to induce the serial dependence in that feature for the subsequent test image to a certain degree^19,55,56^. In the current experiment, participants were asked to look at the fixation point at the center of the adaptor image during the change detection task, and then to report the percept for the subsequent Necker lattice. In such a task, attentional leak to an overall spatial extent of the adaptor from the fixation point may occur to a certain extent while performing the change detection task, leading to the emergence of the positive effect for the upcoming Necker lattice; the current size of the adaptor with the fixation point was sufficiently smaller than a spatial region (the continuity field) in which a prior stimulus attracts a percept of the current stimulus^14^. From this perspective, neural processing underlying the P1 enhancement may be shared with that related to serial dependence.

The exact neural mechanism underlying P1 enhancement remains unclear. Early visual processing within 150 ms following the onset of a visual image is not exclusively consistent with the bottom-up processing (e.g., Ref.^57^), and the fast frontal activity evoked by an image was suggested to regulate early visual activities evoked by the same image, represented by P1 and N1, via top-down processing^47^. In addition, the right dorsomedial prefrontal cortex (dmPFC) was reported to be involved in the generation of the positive bias for a bistable image^9^. Taken together, in the present study, the dmPFC may have sent a top-down signal for early visual processing underlying P1 at a latency of around 120 ms, and such top-down processing may play a role in counteracting the reverse-bias effect. Of note, although paradoxical, our current study demonstrated that P1 enhancement relevant to the weakening of the negative bias was invoked by the adaptor yielding the negative bias; the adaptor invoked two counteractive visual processes for a subsequent image in shaping a percept of the image. The current study may disclose such paradoxical visual mechanisms based on the fast interaction between the bottom-up process and the top-down process at as early as around 120 ms.

Correlation analyses between VEP and the behavioral data were carried out with a focus on the inter-individual differences. Although this analytical approach is powerful to deduce the neural mechanisms underlying behavioral data^31^, it remains unclear how the inter-individual difference in the P1 amplitude occurred in the brain and how this difference correlated with reduction of the reverse-bias effect. A previous fMRI study with a continuously-presented bistable stimulus demonstrated that inter-individual differences in the bistable perception are due to differences in the strength of reciprocal functional connections between a visual area and higher areas; the connectivity was presumed to reflect the iterative interaction between bottom-up and top-down processing^58^. As in this previous study, the correlation found in the current study may capture one facet of inter-individually-variable interactions between the top-down visual process and bottom-up process under an identical experimental scheme across participants.

Our current results showed a significant correlation between the P1 enhancement and the reduction of the reverse-bias effect for the right electrode ROI but not for the left electrode ROI. Neural mechanism relevant to induction of the lateralization is still unclear. Previous studies reported that the right hemisphere was related to perceptual alternation of the bistable visual stimulus (e.g., Refs.^50,59^), and the right frontoparietal areas were proposed to be involved in selection of neuronal events, leading to visual awareness^59^. As described above, the present study was not expected to tap neural mechanism relevant to the perceptual alternation. Nevertheless, the current findings may expand the previous proposition in that the lateralized visual processing is generally relevant to neural processing of visual ambiguity and to reduction of an adaptation-induced perceptual bias.

Differences in stimulation method between the RB and Control conditions may have affected the present results to some extent. In the current experiment, the time period of behavioral tasks for detecting fixation changes was longer in the RB condition than in the Control condition (see Fig. 1). In addition, the Control condition was always followed by the RB condition. Taking these factors into account, it was possible that participants were more fatigued in the RB condition than in the Control condition; such a difference in fatigue may alter the behavioral response between the conditions. As reported above, participants performed the behavioral task of detection of fixation changes in both the RB and Control conditions; the response rate to all fixation changes was nearly or equal to 1 for all participants in both conditions. Thus, fatigue was not an important factor. In addition, as the Control condition preceded the RB condition, overall neural adaptation to visual stimuli in the RB condition was expected to be greater than that in the Control condition. This raised a possibility that the P1 enhancement and N1 diminishment were underestimated and overestimated, respectively. The current main finding that the P1 enhancement is relevant to the reduction of the reverse-bias effect was based on the correlation analyses, which were performed in terms of inter-individual differences. In these analyses, the difference in peak latency/amplitude of VEPs (RB condition–Control condition) and the strength of the reverse-bias effect (differential proportion of the left-facing percept of the Necker lattice, RB condition–Control condition) were respectively calculated for each participant, and these indices were used to calculate correlation coefficients between neural and behavioral data across participants (see “Methods”). In such an analytical procedure, the order of the experimental conditions, which was identical across participants, was orthogonal to outcomes of the correlation analyses. Therefore, the present findings cannot be merely accounted for by potential effects of the order of experimental conditions as they were based on the correlation analyses.

In conclusion, the present study suggests specific early visual processing related to reduction of the reverse-bias effect (a negative bias) by focusing on the P1 enhancement for the test image. Visual processing underlying the P1 enhancement may enable the visual system to pave the way for the positive bias to be more likely to occur even under a sensory condition where the negative bias is expected. In the face of an ever-fluctuating sensory environment, such visual processing may function in a balancing act between the uptake of conspicuous visual information in relation to preceding sensory inputs and the stabilization of what we see over time.

## Data Availability

Data sets processed during the present study are available from the corresponding author on reasonable request.
